# Reducing transfusion utilization for children with sickle cell anemia in sub-Saharan Africa with hydroxyurea: Analysis from the phase I/II REACH trial

**DOI:** 10.1002/ajh.27244

**Published:** 2024-02-08

**Authors:** Alexandra Power-Hays, George A. Tomlinson, Leon Tshilolo, Brígida Santos, Thomas N. Williams, Peter Olupot-Olupot, Luke R. Smart, Banu Aygun, Adam Lane, Susan E. Stuber, Teresa Latham, Russell E. Ware

**Affiliations:** 1 Division of Hematology and Global Health Center, Cincinnati Children’s Hospital Medical Center, Cincinnati, Ohio, USA; 2 Department of Pediatrics, University of Cincinnati, Cincinnati, Ohio, USA; 3 Department of Medicine, University Health Network, Toronto General Hospital, Toronto, Ontario, Canada; 4 Department of Medicine, Centre Hospitalier Monkole, Kinshasa, Congo; 5 Instituto Hematológico Pediátrico, Hospital Pediátrico David Bernardino, Luanda, Angola; 6 KEMRI-Wellcome Trust Programme, Kilifi, Kenya; 7 Mbale Clinical Research Institute, Mbale Regional Referral and Teaching Hospital-Busitema University, Mbale, Uganda; 8 Division of Hematology, Cohen Children’s Medical Center of New York, New Hyde Park, New York, USA

## Abstract

Children with sickle cell anemia (SCA) in Africa frequently require transfusions for SCA complications. Despite limited blood supplies, strategies to reduce their transfusion needs have not been widely evaluated or implemented. We analyzed transfusion utilization in children with SCA before and during hydroxyurea treatment. REACH (Realizing Effectiveness Across Continents with Hydroxyurea, NCT01966731) is a longitudinal Phase I/II trial of hydroxyurea in children with SCA from Angola, Democratic Republic of Congo, Kenya, and Uganda. After enrollment, children had a two-month pre-treatment screening period followed by 6 months of fixed-dose hydroxyurea (15–20 mg/kg/day), 18 months of dose escalation, and then stable dosing at maximum tolerated dose (MTD). Characteristics associated with transfusions were analyzed with univariate and multivariable models. Transfusion incidence rate ratios (IRR) across treatment periods were calculated. Among 635 enrolled children with 4124 person-years of observation, 258 participants (40.4%) received 545 transfusions. The transfusion rate per 100 person-years was 43.2 before hydroxyurea, 21.7 on fixed-dose, 14.5 during dose escalation, and 10.8 on MTD. During MTD, transfusion incidence was reduced by 75% compared to pre-treatment (IRR 0.25, 95% confidence interval [CI] 0.18–0.35, *p* < .0001), and by 50% compared to fixed dose (IRR 0.50, 95% CI 0.39–0.63, *p* < .0001). Hydroxyurea at MTD decreases transfusion utilization in African children with SCA. If widely implemented, universal testing and hydroxyurea treatment at MTD could potentially prevent 21% of all pediatric transfusions administered in sub-Saharan Africa. Increasing hydroxyurea access for SCA should decrease the transfusion burden and increase the overall blood supply.

## 1 | INTRODUCTION

Sickle cell anemia (SCA) is one of the most common causes of hemolytic anemia worldwide, with an estimated 386 000 infants born every year with the disease.^1,2^ Over 75% of these births occur in sub-Saharan Africa, where SCA is a major etiology of severe childhood anemia and where people living with SCA depend on blood transfusions to treat acute and life-threatening complications.^3^ Although blood supplies are often limited, strategies to reduce the transfusion needs of children with SCA have not been widely evaluated or implemented.

Blood is a scarce, vital resource worldwide, and sub-Saharan Africa faces the greatest deficit between blood transfusion demand and safe supply.^4–6^ In Africa, 47% of blood transfusion recipients are children, which is in stark contrast to North America and Europe, where the majority of transfusion recipients are over 60 years old.^6,7^ Although birth prevalence of SCA is 1%–2%, several studies have documented that ~30% of pediatric transfusion recipients and 18% of transfusion recipients of all ages in sub-Saharan Africa have SCA.^1,7–9^ Furthermore, untreated children with SCA are 20 times more likely to require recurrent blood transfusions than those without SCA.^10^ Due to the lack of universal testing for SCA in most African countries, and exclusion of patients with known SCA from many studies on blood transfusion use, these statistics are likely underestimates.

This heavy reliance on blood transfusions for management of sickle cell complications confers risks to the individual recipients, including blood-borne infection transmission, transfusion reactions, erythrocyte alloimmunization, and iron overload, but also contributes to the overall blood supply deficit in countries with high SCA prevalence in Africa.^3^ Reducing transfusion rates in SCA would not only improve the health of individuals living with this disease, but also could be an under-recognized strategy to decrease blood scarcity in regions with high SCA prevalence in Africa and globally.

Affected individuals with SCA often require blood transfusions as acute interventions for sickle-related complications such as stroke, acute chest syndrome (ACS), transient aplastic crisis, or acute splenic sequestration.^2,3^ Many of these SCA complications are preventable with hydroxyurea, an oral, disease-modifying therapy approved and recommended for the treatment of SCA in the United States, Europe, and Africa.^11^ Hydroxyurea is approved to reduce the need for blood transfusions in people with SCA based, in part, on a prospective cohort study in Europe (ESCORT HU, NCT02516579) that found a 21% reduction in blood transfusions after starting hydroxyurea.^12^ Several studies of hydroxyurea for the treatment of SCA corroborate this reduction in transfusions, but all report transfusion rate as a secondary outcome, and none provided detailed analysis of the transfusion indications, the effect of other illnesses, specifically malaria, or the effects of hydroxyurea-dosing strategy on this critical outcome.^13–16^

The Realizing Effectiveness Across Continents with Hydroxyurea (REACH, NCT01966731) trial is a phase I/II, open-label trial of the feasibility, safety, and benefits of hydroxyurea therapy for children with SCA in Angola, the Democratic Republic of Congo (DRC), Kenya, and Uganda.^17^ Hydroxyurea was found to be safe and effective at reducing the morbidity and mortality associated with SCA; furthermore, treatment was associated with a 67% decrease in blood transfusions in the initial 30-month analysis. Because the reduction of blood transfusion use in children with SCA has implications both for individual health and for broader public health, we examined blood transfusion rates and indications in the REACH trial over an extended observation period. Our aims include characterizing transfusions (rates, indications, and practices) before and during hydroxyurea treatment and identifying hydroxyurea-dosing strategies that lead to the greatest benefits in sub-Saharan Africa.

## 2 | METHODS

### 2.1 | REACH trial overview

A detailed description of the study design, analysis, and primary results have been published.^17^ Patients were recruited from sickle cell clinics in Luanda, Angola; Kinshasa, Democratic Republic of Congo; Kilifi, Kenya; and Mbale, Uganda. Children were eligible if they had a diagnosis of SCA, genotype HbSS, and were between the ages of 1 and 10 years. Exclusion criteria were recent hydroxyurea treatment, blood transfusion within 30 days (temporary exclusion), and presence of another chronic illness (human immunodeficiency virus [HIV], tuberculosis, cancer). Throughout the pre-treatment and treatment periods, participants received local standard-of-care therapy including age-appropriate vaccines, penicillin prophylaxis for those under 5 years old, and malaria prophylaxis as per national guidelines. Patients received proguanil in Kenya, sulfadoxine/pyrimethamine in Uganda, and no malaria prophylaxis in Angola or DRC. All participants in the REACH trial and all transfusions that occurred during the trial are included in this analysis. Written informed consent and assent when applicable were obtained from all patients or guardians. The study was approved by the Institutional Review Boards of Cincinnati Children’s Hospital Medical Center and all four international clinical performance sites.

### 2.2 | Hydroxyurea treatment

All participants underwent a 2-month, pre-treatment, screening period followed by open-label hydroxyurea treatment for the remainder of the study. For the first 6 months of treatment, participants were prescribed a fixed dose of hydroxyurea ranging from 15 to 20 mg/kg/day. Participant doses were escalated during months 6–24 of study treatment to maximum tolerated dose (MTD), which maximizes induction of protective fetal hemoglobin (HbF) with mild myelosuppression, targeting an absolute neutrophil count (ANC) less than 4.0 × 10^9^/L and platelet count less than 150 × 10^9^/L without inducing myelotoxicity, which is defined as an ANC less than 1.0 × 10^9^/L or platelet count less than 80 × 10^9^/L. After 24 months, participants continued on hydroxyurea with dose adjustments based on weight gain or laboratory values. Hydroxyurea for the REACH trial was donated by Bristol-Myers Squib, who had no access to the data, no oversight of the analyses, and did not review the manuscript before submission.

### 2.3 | Transfusion data collection

Transfusions were recorded prospectively in the REACH REDCap database. The decision to transfuse was made independently by local clinicians, and donated blood units were collected, processed, screened, and distributed in accordance with each country’s national policy. All transfusions of whole blood or packed red blood cells (pRBC) across the four clinical sites were included in this analysis. Descriptive data about the transfusion events included transfusion date, transfusion location (REACH site vs. alternate site), clinical indications, type of blood product (whole blood or pRBC), volume transfused in mL or units, and when available, blood group and cross-match results, the pre-transfusion and post-transfusion hemoglobin values and supplemental diagnostic test results. If recognized, transfusion reactions were recorded as clinical Adverse Events. The clinical indications were specified by the local clinicians and were cross-referenced with other study data including malaria assessments, laboratory values, and adverse events. Although multiple clinical indications could be selected during the trial, a principal indication was identified for analysis (Figure S1).

Demographic data were also collected from the database, including date of birth, sex, G6PD and alpha-thalassemia status, and country of residence. Physical exam and laboratory parameters at study enrollment, at each scheduled visit, and at presentation for transfusion were recorded if available. Transfusions were categorized based on the treatment period of the participant when the transfusion occurred; these categories include pre-treatment (screening), fixed-dose (months 0–6), dose escalation (months 7–24), and treatment at MTD (months 24 onward). If on treatment, the duration that the participant had received hydroxyurea was recorded.

### 2.4 | Statistical analysis

Descriptive statistics were used to characterize participants’ baseline characteristics in each of three groups according to their transfusion rates over the trial (0, 1–25, and >25 transfusions per 100 person-years). Analysis of variance and chi-squared tests were used to compare characteristics between the groups, as appropriate. The incidence rate ratios (IRR) comparing transfusion rates across treatment periods were estimated in a Poisson GEE model with clustering by participant and the logarithm of time at risk in each treatment period as the offset. The Andersen-Gill model for recurrent events was used to estimate the relationship between the risk of transfusion and both fixed baseline and time-varying participant characteristics, with laboratory values lagged by 30 days; hazard ratios (HR) are shown for both univariate and multivariable models. *p*-Values less than .05 were considered statistically significant. All analyses were carried out using R (Vienna, Austria).^18^

## 3 | RESULTS

Transfusions were recorded from July 2014 to January 2023 for a total of 4124 person-years. A total of 635 children were enrolled in the REACH trial; 606 completed screening and began receiving hydroxyurea.

### 3.1 | Transfusion characteristics and risk factors

A total of 545 transfusions were administered during the trial to 258 participants (Table 1). There was variation in the type of blood product administered by country. The majority of transfusions were whole blood in Kenya (100%) and in Uganda (72.2%); in contrast, pRBC were more commonly administered in Angola (99.1%) and DRC (94.0%). Although the mean hemoglobin at baseline was low across all sites, 7.3 ± 1.1 g/dL at enrollment, children who were transfused developed severe anemia prior to transfusion, with the mean pre-transfusion hemoglobin concentration across all sites as 4.4 ± 1.3 g/dL.

On treatment, 236 participants (37.1%) were administered transfusions, and 133 children (20.9%) received two or more transfusions. By univariate analysis, those who subsequently had higher transfusion rates were younger, had lower hemoglobin and fetal hemoglobin (HbF %), and more frequently had palpable spleens at enrollment (Supplemental Table). There were no statistically significant effects of sex, history of splenectomy, G6PD deficiency, or alpha thalassemia on transfusion rates.

In the multivariable Andersen-Gill model for recurrent transfusions (Table 2), palpable splenomegaly at enrollment was a strong predictor of receipt of a transfusion (HR 1.61, 95% confidence interval [CI] 1.32–1.98, *p* = .0008). Additional risk factors for a transfusion during treatment included a transfusion during screening (HR 1.81, 95% CI 1.35–2.41, *p* = .0047), and an elevated absolute reticulocyte count in the month prior to transfusion (HR 1.20 per 2-fold increase in count, 95% CI 1.05–1.38, *p* = .0289). Protective factors included older age at enrollment (HR 0.93 per 1 year increase, 95% CI 0.89–0.97, *p* = .0076), and higher hemoglobin (HR 0.63 per 1 g/dL increase, 95% CI 0.59–0.67, *p* < .0001).

### 3.2 | Transfusion use before and during hydroxyurea treatment

**FIGURE 1:**
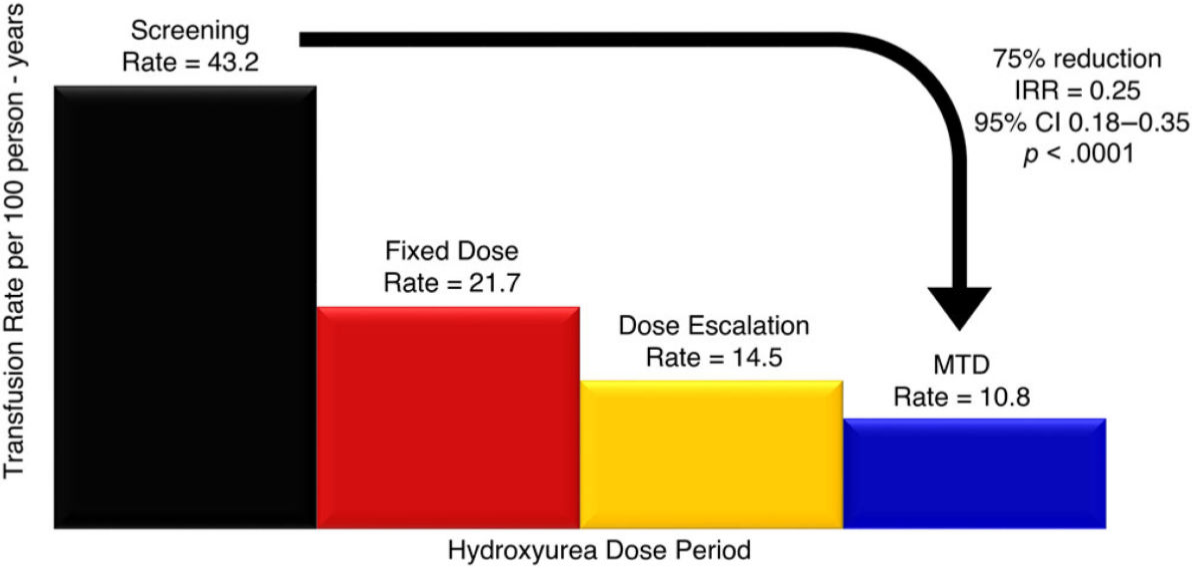
Transfusion rate per 100 patient-years administered to children with sickle cell anemia based on hydroxyurea dose period The transfusion rate per 100 person-years observed during each hydroxyurea-dosing period is presented. During the pre-treatment screening period, the transfusion rate per 100 person-years was 43.2, which decreased to 21.7 on moderate, fixed dose (15–20 mg/kg/day), 14.5 during dose escalation, and to 10.8 on maximum tolerated dose (MTD). Treatment on MTD leads to a 75% reduction in transfusions compared with the pre-treatment period (incidence rate ratio [IRR] 0.25, 95% confidence interval 0.18–0.35, *p* < .0001), and a 50% reduction compared with treatment on the moderate, fixed dose.

The transfusion rate prior to starting hydroxyurea was 43.2 per 100 person-years (Figure 1). Treatment with fixed dose hydroxyurea decreased this rate by half to 21.7 per 100 person-years (IRR 0.50, 95% CI 0.34–0.73, *p* = .004), and during dose escalation to MTD the rate decreased further to 14.5 per 100 person-years (IRR 0.34, 95% CI 0.23–0.48, *p* < .0001). During hydroxyurea treatment at MTD there were 10.8 transfusions per 100 person-years; this represents a 75% lower transfusion rate compared with the pre-treatment period (IRR 0.25, 95% CI 0.18–0.35, *p* < .0001) and a 50% reduction compared with treatment on moderate, fixed dosing (IRR 0.50, 95% CI 0.39–0.63, *p* < .0001).

### 3.3 | Transfusion indications

Only 51 (9.3%) transfusion events did not have an indication recorded. Of the remaining 494 transfusions, the most common indications for transfusions during REACH were anemia (45.1%) and anemia due to malaria (27.7%, Table 2). Another 13.0% were for SCA-related complications: stroke or neurologic event (2.9%), ACS or respiratory event (2.4%), acute splenic sequestration (4.4%), or pain (3.3%).

With hydroxyurea treatment at MTD, there was an 83% reduction in transfusions for anemia (IRR 0.17, 95% CI 0.18–0.35, *p* < .0001); 54% reduction in transfusions for anemia due to malaria (IRR 0.46, 95% CI 0.22–0.98, *p* = .0428); and 72% reduction for transfusions to treat acute SCA-related complications (IRR 0.28, 95% CI 0.09–0.85, *p* = .0246) compared with the pre-treatment period (Table 3). Although the rate of transfusions for anemia decreased across treatment periods, the mean pre-transfusion hemoglobin concentration did not differ significantly, 4.7 ± 1.0 g/dL during screening, 4.6 ± 1.4 g/dL during fixed-dose treatment, 4.5± 1.2 g/dL during dose escalation, and 4.4 ± 1.3 g/dL during treatment at MTD ( *p* = .59).

The cumulative number of transfusions per child in DRC was 1.75 over 8 years (Figure S2A). In comparison to DRC, the risk of transfusion in the other countries was lower: Angola (HR 0.51, 95% CI 0.41–0.64, *p* = .0003), Kenya (HR 0.24, 95% CI 0.18–0.32, *p* < .0001), and Uganda (HR 0.39, 95% CI 0.30–0.49, *p* < .0001). Forty percent of the transfusions in DRC were for anemia in the setting of malaria compared with 10.6% in Angola, 16.4% in Kenya, and 23.6% in Uganda. A greater than 3-fold increase in the cumulative transfusions for anemia due to malaria in DRC explains much of the difference in transfusion rates between countries (Figure S2B).

## 4 | DISCUSSION

Our extended analysis of the REACH cohort finds that the overall transfusion rate is high among children with SCA in sub-Saharan Africa not receiving hydroxyurea, and suggests that 75% of blood transfusions administered to this population could be prevented with hydroxyurea treatment at MTD. Most transfusions were administered for anemia as the specific clinical indication, with younger age at enrollment, lower hemoglobin, and palpable splenomegaly identified as risk factors for requiring a blood transfusion. Recognition and implementation of strategies to prevent blood transfusions in this patient population could improve the health of individuals with SCA, by avoiding the potential risks of transfusion-related complications and could reduce the demand for safe blood, a scarce resource.

It is critical to recognize the high reliance on blood transfusions among children with SCA who are not receiving hydroxyurea. In REACH, the transfusion rate prior to starting on hydroxyurea was 43.2 transfusions per 100 person-years across study sites. A high rate of blood transfusion administration to children with SCA not receiving hydroxyurea has been reported in other trials and real-world studies,^7,10,19^ and even exceeded 100 transfusions per 100 person-years in a region in Uganda with high malarial prevalence.^20^ Due to the lack of widespread, universal testing for SCA, most transfused children may not have a proper diagnosis of SCA; nonetheless, without intentional SCA-directed therapy their acute medical complications are being treated with sporadic blood transfusions.

It is also important to remember that blood transfusions are not benign interventions and pose numerous risks to transfusion recipients. Recurrent transfusions can contribute to iron overload, erythrocyte alloimmunization, and non-immune hemolytic transfusion reactions. Despite universal screening policies, transfusion-transmissible infection rates in low-income countries are not insubstantial (1.1% HIV, 1.0% hepatitis C, 3.7% hepatitis B).^6^ In REACH, two children acquired HIV through separate blood transfusions during the trial.

Hydroxyurea decreased transfusion requirements in children with SCA within sub-Saharan Africa, a finding which has been demonstrated in previous studies in high- and low-income countries.^12–15^ Our analysis provides further evidence of this reduction with extended longitudinal analysis and highlights the importance of hydroxyurea dose, with the greatest reduction in transfusions occurring with treatment at MTD. Hydroxyurea treatment at a low or moderate dose has been proposed as a cost-saving approach or to decrease the need for laboratory monitoring.^21^ Our results demonstrate that treatment with a fixed, moderate dose of hydroxyurea (~20 mg/kg/day) reduces the blood transfusion rate in this population by 50%, but treatment at MTD with dose optimization (mean ~ 24.0 mg/kg/day) was associated with a 75% reduction over the untreated period, and an additional 50% reduction in blood transfusion rate over treatment at fixed, moderate dose. Furthermore, treatment at MTD has also been shown to provide better protection against anemia, stroke, pain, and malaria.^12,16,17^ A recent cost-effectiveness model estimates that in Uganda, children with SCA taking hydroxyurea at MTD would receive a median of 3.5 units of blood during childhood (from diagnosis through age 18 years) compared with 5.6 units of blood on hydroxyurea at fixed-dose, and 14.3 units without treatment.^22^

The logistical challenges of traditional dose escalation to MTD are not trivial, and strategies are needed to decrease the burden of achieving MTD, such as guaranteeing the presence of reagents for complete blood counts and assisting families with travel to clinic.^23–25^ Pharmacokinetic (PK)-guided dosing may reduce laboratory monitoring, and several prospective trials are underway that will evaluate the feasibility and benefits of PK-guided hydroxyurea dosing in Africa, including the Alternative Dosing And Prevention of Transfusions (ADAPT, NCT05662098) trial in Uganda; a follow-on study of the Stroke Prevention with Hydroxyurea Enabled through Research and Education (SPHERE, NCT03948867) in Tanzania; the Prioritizing Utilization and Safety of Hydroxyurea Using Precision (PUSH-UP, NCT05285917) trial in Angola; and an extension of the REACH trial.

To achieve the reduction in transfusions observed in the REACH trial across a larger population of children with SCA, accurate diagnosis, comprehensive care, and hydroxyurea are all necessary, but we note that hydroxyurea treatment was the primary modifier of transfusion utilization. Comprehensive care with anticipatory guidance, infection prevention, and supportive care have been shown to reduce mortality in SCA,^26^ but children in the REACH trial received comprehensive care during both the pre-treatment and treatment periods. They were treated by the same clinicians during the pre-treatment and treatment periods, and the mean pre-transfusion hemoglobin levels were not statistically different between periods. Therefore, the change in transfusion use cannot be accounted for by changes in provider practice or supportive care measures and should be attributed to treatment with hydroxyurea.

In terms of transfusion indications, nearly half of the transfusions in the REACH trial during screening and on treatment were for anemia, and the mean pre-transfusion hemoglobin in the study was 4.4 g/dL. The World Health Organization defines severe anemia as less than 6 g/dL, but the Transfusion and Treatment of Severe Anemia in African Children Trial (TRACT) concluded that African children with uncomplicated severe anemia (Hb 4–6 g/dL without other signs of clinical severity) could be monitored clinically rather than transfused solely for the low hemoglobin.^19,27^ Because children with SCA may have a baseline hemoglobin around 6–7 g/dL, a well-appearing child should not receive a transfusion for a hemoglobin threshold alone, but an acute drop in hemoglobin due to increased hemolysis or malarial infection, could quickly result in critical illness.^3^ In REACH, hydroxyurea treatment at MTD led to an 83% reduction in transfusions for anemia, likely by increasing the hemoglobin level of each child to provide a buffer in the event of an acute drop and by decreasing hemolysis from intracellular sickling due to increased HbF%, the main mechanism of disease-modification by hydroxyurea.

Another 27% of transfusions in the trial were for anemia due to malarial infection, and there was a 3-fold increase in transfusions for this indication in DRC compared with the other study sites. This is likely due to differences in malaria prevalence, use of bed nets and chemoprophylaxis per national guidelines, or parasite resistance.^28^ SCA affords protection against high-density malarial infections, but even low-density infections can precipitate a catastrophic drop in hemoglobin in children with SCA.^8^ As such, those receiving a blood transfusion in high-prevalence areas should be tested for SCA, even if they are positive for malaria. Hydroxyurea treatment at MTD decreased malaria infections in children with SCA by 65% in the REACH trial,^17^ and here we show that it also decreases transfusions for anemia in the setting of malaria.

In addition to the individual health benefits, there are substantial, potential, public health benefits to treating those with SCA; reducing transfusion utilization is crucial to addressing the blood scarcity in sub-Saharan Africa. If 29% of pediatric blood transfusion recipients have SCA in the region,^8,29^ then universal testing and hydroxyurea treatment at MTD could potentially prevent 75% of those transfusions, which represents ~21% of all pediatric transfusions administered in sub-Saharan Africa. Local, national, and international efforts to improve access to safe blood for transfusions in Africa must include efforts to reduce the transfusion burden among those with SCA through universal diagnosis and hydroxyurea treatment for the benefit of individuals with the disease as well as for the health of the general population. With such a high burden of transfusions for SCA in this study, until universal diagnosis is available, children receiving a transfusion in high-prevalence regions should be tested for SCA and offered hydroxyurea once the diagnosis is established.

Despite the overwhelming benefits of hydroxyurea in reduction in stroke, pain, malaria, and death, most African children with SCA do not have access to this potent and life-saving disease-modifying therapy. The cost of hydroxyurea is one major barrier cited to widespread uptake.^22,30,31^ The countries in the REACH trial have varying degrees of publicly available basic health care, but none currently include guaranteed hydroxyurea for people with SCA. Published prices for one daily dose of hydroxyurea of 500 mg range from US $0.13 (Nigerian manufacturer)^30^ to $0.20 (imported into Uganda) to $0.91 (retail pharmacy in Tanzania).^31^ The cost of laboratory monitoring to determine MTD and the tangible and opportunity costs of traveling to clinic for laboratory monitoring must be included in this consideration as well. The costs of the medication and the required monitoring are likely not affordable for most families, but as part of a comprehensive program, hydroxyurea escalated to MTD has been modeled to be cost-effective at reducing blood transfusions at a price point of over $14 (USD in 2023) per unit of blood.^22^ This is about one-tenth of the published cost of processing one unit of blood from a centralized system in Zimbabwe (reported at $130.94, USD in 2015).^32^ Investment in access to hydroxyurea and laboratory monitoring is likely to reduce clinical complications for the benefit of individuals with SCA, and to reduce blood transfusions for public health and economic benefit of the country.

We recognize some limitations to this analysis. First, the REACH trial was not randomized, but the treatment periods allow a valid comparison. Evaluations of the transfusion indications were limited to information provided by the treating clinicians. With more accurate, prospective capture of transfusion indications, additional information could be obtained, such as ensuring a confirmatory hemoglobin level, reticulocyte count, iron status, and malaria testing to elucidate the causes of anemia. Additionally, only transfusions in REACH that were completed were collected and described; therefore, any intended transfusions that were unable to be administered during high utilization periods due to stockouts of blood, might introduce a selection bias.

In conclusion, the REACH trial shows a significant reduction in blood transfusion utilization in children with SCA on hydroxyurea therapy, particularly when administered at an optimal dose. Globally, Africa has the highest burden of SCA and the greatest shortage of safe blood. Treating SCA should be an individual health and public health priority in sub-Saharan Africa, due to both the immense and preventable suffering of affected individuals with SCA and the health system burdens that exist due to extensive and preventable blood transfusion utilization among untreated individuals with this disease.

## Supplementary Material

2

## Figures and Tables

**TABLE 1:** Transfusion characteristics presented by country and overall.

	Angola	DRC	Kenya	Uganda	Overall
Number of transfusions	123	251	61	110	545
Number of participants transfused	55	98	35	70	258
Blood product					
Recorded, *n* (%)	116 (94.3)	199 (79.3)	61 (100.0)	108 (98.2)	484 (88.8)
Packed RBC, *n* (%)	115(99.1)	187 (94.0)	0 (0.0)	30 (27.8)	332 (68.6)
Whole blood, *n* (%)	1 (0.9)	12 (6.0)	61 (100.0)	78 (72.2)	152 (31.4)
Pre-transfusion hemoglobin (g/dL)					
Recorded, *n* (%)	75 (61.0)	126 (50.2)	60 (98.4)	72 (65.5)	333 (61.1)
Pre-transfusion Hb (mean, SD)	4.3 (1.5)	4.6 (1.1)	4.0 (1.1)	4.6 (1.4)	4.4 (1.3)
Primary indication, *n* (%)					
Anemia	74 (60.2)	82 (32.7)	39 (63.9)	51 (46.4)	246 (45.1)
Malaria	13 (10.6)	102 (40.6)	10 (16.4)	26 (23.6)	151 (27.7)
Neurological event	9 (7.3)	0 (0.0)	2 (3.3)	5 (4.5)	16 (2.9)
Respiratory event	6 (4.9)	3 (1.2)	0 (0.0)	4 (3.6)	13 (2.4)
Splenic sequestration	6 (4.9)	8 (3.2)	6(9.8)	4 (3.6)	24 (4.4)
Pain	0 (0.0)	2 (0.8)	0 (0.0)	16 (14.5)	18 (3.3)
Perioperative	13 (10.6)	2 (0.8)	4 (6.6)	2 (1.8)	21 (3.9)

*Note*: There was a difference across countries in blood product most commonly transfused. Across all four study sites, participants were severely anemic prior to transfusion, and anemia, not otherwise specified, was the most common indication for transfusion followed by anemia in the setting of malaria. Transfusions for sickle cell anemia-related complications were evenly divided into those for neurological events, such as stroke, respiratory event, including acute chest syndrome, splenic sequestration, and pain.

Abbreviations: DRC, Democratic Republic of Congo; packed RBC, packed red blood cells.

**TABLE 2:** Multivariable Andersen-Gill model hazard ratios, 95% confidence intervals, and *p*-values.

	Hazard ratio	95% confidence interval	*p*-Value
Hemoglobin (per 1 g/dL increase)	0.63	0.59–0.67	<.0001
HbF (per 10% increase)	0.93	0.83–1.03	.2970
ARC (per 2-fold increase)	1.20	1.05–1.38	.0289
Age (per 1 year increase)	0.93	0.89–0.97	.0076
Sex (male vs. female)	1.02	0.85–1.22	.8798
Spleen at enrollment			
Non-palpable (reference)	-	-	-
History of splenectomy	0.61	0.22–1.64	.2912
Palpable splenomegaly	1.61	1.32–1.98	.0008
Transfusion during screening (vs. none)	1.81	1.35–2.41	.0047

*Note*: Increased hemoglobin and age at enrollment were protective against transfusion, while increased ARC, palpable splenomegaly at enrollment, and a transfusion during screening increased risk of a transfusion during treatment. Laboratory values are 30-day time lagged.

Abbreviations: ARC, absolute reticulocyte count; HbF, fetal hemoglobin.

**TABLE 3:** Changes in the overall transfusion rate and by clinical indication based on hydroxyurea treatment dosing phase are presented with incidence rate ratios (IRR) and 95% confidence intervals (CI) comparing the transfusion rates in each dosing phase to the screening period as the reference.

Dose phase	Screening	Fixed dose	Dose escalation	MTD
Study month	−2 to 0	0–6	7–24	>24
Patient-years	111	300	877	2836
Dose, mean (SD) in mg/kg/day	-	17.6 (2.4)	21.7 (5.0)	24.0 (5.4)

Transfusions	Rate	Rate	IRR (CI)	*p*-Value	Rate	IRR (CI)	*p*-Value	Rate	IRR (CI)	*p*-Value
All indications	43.2	21.7	0.50 (0.34–0.73)	.0004	14.5	0.34 (0.23–0.48)	<.0001	10.8	0.25 (0.18–0.35)	<.0001
Anemia	27.9	9.3	0.34 (0.20–0.57)	.0001	5.7	0.2 (0.13–0.33)	<.0001	4.8	0.17 (0.12–0.26)	<.0001
Malaria	8.1	4.7	0.58 (0.23–1.44)	.2397	2.5	0.31 (0.13–0.73)	.0078	3.7	0.46 (0.22–0.98)	.0428
Sickle-related complication	3.6	4.3	1.21 (0.39–3.76)	.7467	2.9	0.79 (0.27–2.29)	.6679	1.0	0.28 (0.09–0.85)	.0246

*Note*: The transfusion rate decreases by 50% (IRR 0.50) with treatment on moderate, fixed-dose, by 66% (IRR 0.34) during dose escalation, and by 75% (IRR 0.25) with hydroxyurea treatment at maximum tolerated dose. There is a similar reduction in transfusions for anemia, not otherwise specified, and for anemia in the setting of malaria. There is a 72% in transfusions for sickle cell-related complications reduction (IRR 0.28) with treatment at MTD compared with the pre-treatment period.

Abbreviation: MTD, maximum tolerated dose.

## Data Availability

Requests for deidentified participant data which underlie the results reported in this manuscript may be directed to the corresponding author during a period beginning 9 months following publication and ending 36 months following publication. Requests must include an institutional review board-approved proposal. Deidentified data will be made available following approval by an internal review committee and completion of a data sharing agreement between all parties.
