# Supplementary material for: Reducing transfusion utilization for children with sickle cell anemia in sub-Saharan Africa with hydroxyurea: Analysis from the phase I/II REACH trial
Source: Am J Hematol. Author manuscript; Available in PMC 2025 Apr 1. (PMC11289910; doi:10.1002/ajh.27244)
Supplement: 2 [file NIHMS2001882-supplement-2.pdf]

REACH Trial investigator teams and key contributors

1) Centre Hospitalier Monkole, Kinshasa, Democratic Republic of Congo

   Principal Investigator: Léon Tshilolo, 156 participants

   Site personnel: Michael Kasongo, Sylvain Fazili, Nancy Madingo, Jonathan Kukila, Christian Babaku, Didier Mbuyi, Gisèle Mankande, Landry Kipepe, Armelle Lubadika and Merveilles Mbombo;

2) KEMRI Wellcome Trust Research Program, Kilifi, Kenya

   Principal Investigator: Thomas N. Williams, 151 participants

   Site personnel: George Mochamah, Maurine Maoni, Alex Macharia, Gideon Nyutu, Jimmy Shangala, Ruth Mwarabu, Metrine Tendwa, Jacob Golijo, Johnstone Makale, Brian Tawa and Kathryn Maitland;

3) Hospital Pediátrico David Bernardino, Luanda, Angola

   Principal Investigator: Brígida Santos, 150 participants

   Site personnel: Luis Bernardino, José Luis Reis da Fonseca, Lourenço Nassesa, Darío Adão de Oliveira André, Leydma Cuhna, Kembo Andre, Rui Pascal and Vysolela de Oliveira;

4) Mbale Clinical Research Institute, Mbale, Uganda:

   Principal Investigator: Peter Olupot-Olupot, 149 participants

   Site personnel: George Paasi, Linus Ochen, Erayu Godfrey Bonface, Timothy Kirwa, Faizo Machoka, Alex Sande, Felix Opio, and Beatrice Amede;

Consultants and Supporting Staff: George Tomlinson, University of Toronto, Toronto, Canada; Banu Aygun, Cohen Children’s Medical Center, New Hyde Park, New York;

Data Management Center, Division of Hematology, Department of Pediatrics, Cincinnati Children's Hospital, USA: Teresa S. Latham, Adam Lane, Justin McAdams, Jan Englehart, Amanda Pfeiffer, Rebecca Geer, Craig Slusher and John Boesing;

Medical Coordinating Center, Division of Hematology, Department of Pediatrics, Cincinnati Children's Hospital, USA: Russell E. Ware, Luke R. Smart, Susan E. Stuber, Charles Quinn, Thad Howard, and Kathryn McElhinney.

Protocol Amendments

The REACH protocol has had 4 amendments on the following dates:

1) 4/11/2014 (1.1): added two objectives: exploration of the genetic basis for any observed inter-patient variability in the clinical or laboratory response to hydroxyurea and economic cost of providing hydroxyurea therapy; common study end date will be 4 years the first patient treatment commences; sample size increased from 3 sites to 4 sites; dosing calculator added; serious adverse reporting clarified.

2) 11/23/2014 (2): one co-investigator has been removed; four clinical sites have been identified as the initial clinical sites; changed enrollment will halt after 60 children are enrolled at each site for safety analyses instead of 53 to account for drop-out or discontinuation.

3) 6/2/2016 (3): added evaluation of pharmacokinetic parameters for analysis of response to hydroxyurea; added patients may need further dose adjustments during month 12-48 based on weight and blood counts.

4) 8/4/2017 (4): added study treatment will be provided until the participants reach age 18 years and added Tanner staging, menarche and pregnancy testing, urine for kidney function, and TCD assessments; added pregnancy avoidance and procedures for participants of child-bearing age.

REACH Dosing Phases

Screening (2 months)

↓

Fixed dose (0-6 months)
Hydroxyurea at 17.5 ± 2.5 mg/kg/day

↓

Dose escalation (7-24 months)
Escalate hydroxyurea dose to MTD

↓

MTD/Dose optimisation phase (after 24 months)
Maintain/optimise dose based on weight gain and laboratory parameters

REACH Hydroxyurea Dosing Calculator

The REACH dosing calculators have proven to be a valuable tool to managing hydroxyurea dosing throughout the REACH trial. In conjunction with ongoing review by the REACH Hydroxyurea Consultant and study monitoring teams, REACH site teams are able to use these tools to both maximize the accuracy of hydroxyurea dosing and optimize treatment benefit for study participants.

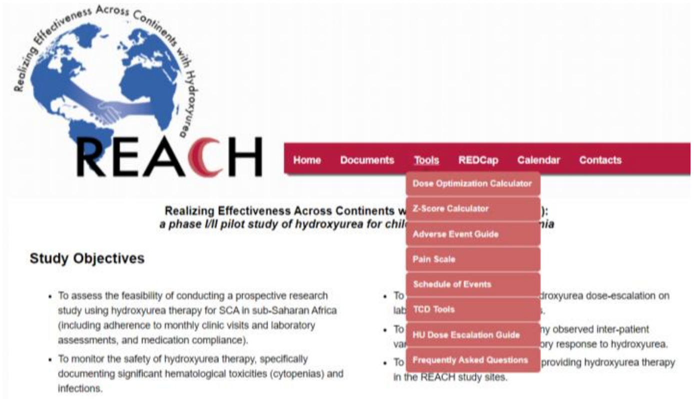

The REACH study website contains the hydroxyurea dosing calculators, which assist site personnel in determining any potential hematological toxicity and in calculating the appropriate hydroxyurea dose during the MTD and dose optimization phase. The dosing calculator is maintained by the Data Management Center (DMC) in collaboration with the Medical Coordinating Center (MCC) and Hydroxyurea Consultant. Fixed and Dose Escalation calculators were provided to sites during earlier phases of the study to guide dosing of hydroxyurea, and currently a Dose Optimization calculator is in use to ensure that REACH participants maintain a proper dose to maintain maximum clinical and hematological benefits. The calculator can be accessed by navigating to the Tools section of the REACH web site.

Toxicity Tool

Before using any of the dosing calculators, the results of a recent complete blood count (including the absolute neutrophil count and absolute reticulocyte count) must be available. If these results reveal no hematological toxicities, then the calculator will automatically move to the dosing screen.

Study teams enter the laboratory results into the Toxicity Tool at each visit prior to dispensing hydroxyurea.

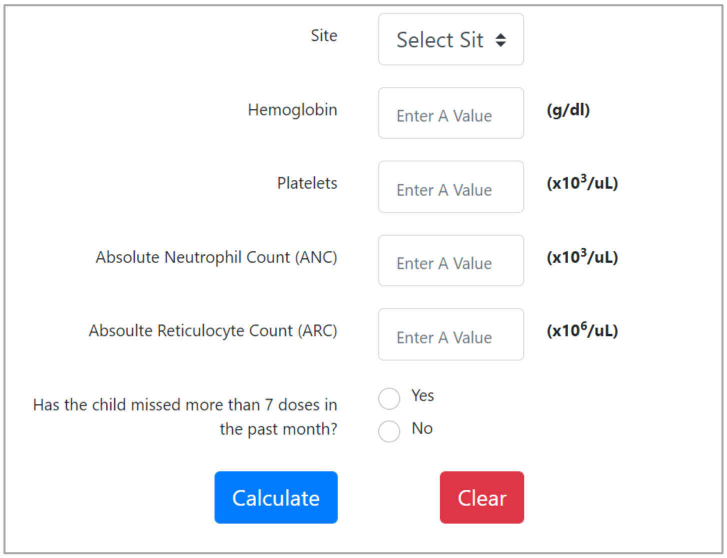

The medication adherence check is based on parent/participant report, to prevent dose increases when >7 doses have been missed

Dose Hold for Hematological Toxicity

If laboratory values document any hematological toxicity, the calculator will identify that the hematological criteria for dosing are not met, and will indicate this with a message directing the site to re-check labs in one week.

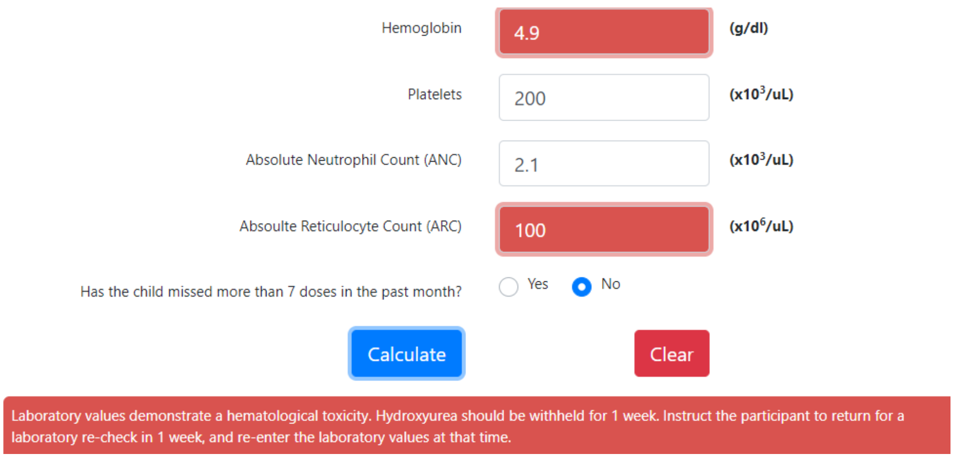

Dosing Screen

The Dose Optimization Calculator is used at all scheduled interval visits to determine if laboratory values are adequate for dose continuation and to determine the hydroxyurea dose, which will likely be unchanged unless there is a significant weight change or a change in lab trends that indicate a small change for dose optimization is needed. The Dose Optimization Calculator will use laboratory results to determine if dose escalation is recommended, and will use the participant's weight and previous dose to provide the new recommended dose. If laboratory values are adequate and the dosing screen is shown, the participant's weight (to the nearest 0.1 kilogram, for example 21.3 kg) and current dose are entered into the calculator. After the participant's weight is entered, the "Calculate" button is pressed to provide automatically the new recommended hydroxyurea dose and the number and size of the capsules that should be dispensed.

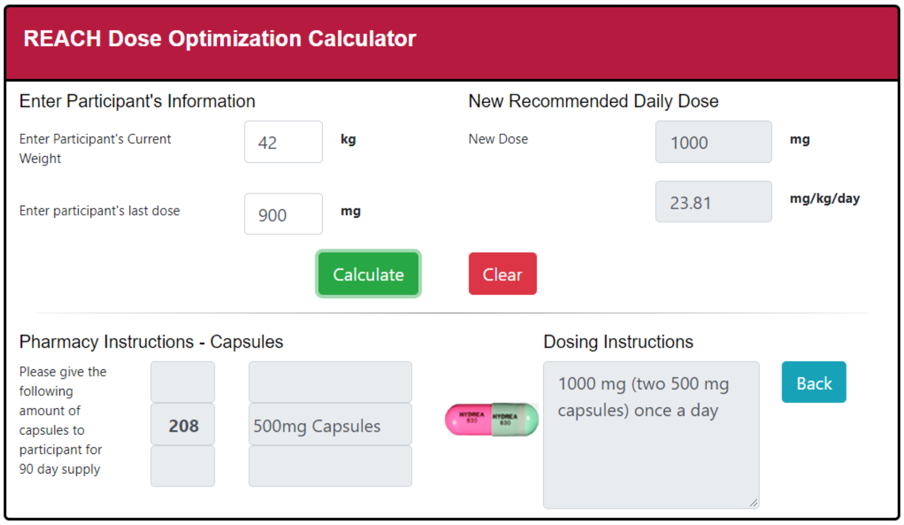

Retention in the REACH trial

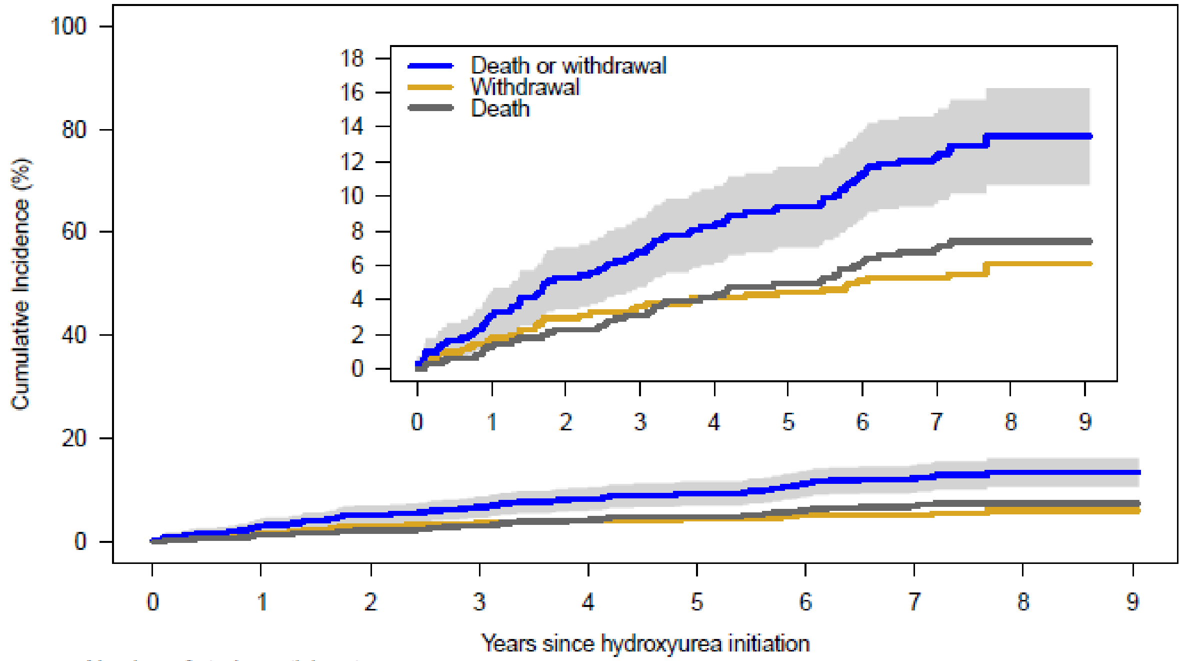

	0	1	2	3	4	5	6	7	8	9
Number of study participants	606	587	574	565	556	549	538	428	235	4
Cumulative number of study withdrawals	0	11	18	22	25	27	31	32	35	35
Cumulative number of deaths	0	8	14	19	25	30	37	42	44	44

	Initiation	Month 6	Year 2	Year 4	Year 6	Year 8	Latest
**Number (N)**	606	591	574	556	538	238	522
**Dose (mg/kg/day)**	17·5 ± 1·9	17·0 ± 2·2	22·2 ± 5·2	23·5 ± 4·9	26·5 ± 5·5	27·0 ± 5·3	28·2 ± 5·2
**Hb (g/dL)**	7·3 ± 1·1	8·1 ± 1·3	8·4 ± 1·3	8·3 ± 1·3	8·2 ± 1·5	8·4 ± 1·5	8·5 ± 1·5
**MCV (fL)**	77 ± 9	85 ± 10	91 ± 13	93 ± 14	95 ± 15	98 ± 15	99 ± 16
**HbF (%)**	10·9 ± 6·8	19·3 ± 8·7	24·1 ± 9·8	22·1 ± 9·4	23·0 ± 9·3	22·9 ± 9·0	23·3 ± 9·5
**ANC (x 10^9/L)**	6·8 ± 3·0	5·3 ± 2·7	4·2 ± 2·1	4·1 ± 2·3	3·8 ± 2·3	3·6 ± 1·9	3·6 ± 2·2
**ARC (x 10^9/L)**	344 ± 147	222 ± 90	183 ± 76	175 ± 80	184 ± 101	193 ± 122	211 ± 136
**Platelets (x 10^9/L)**	411 ± 171	381 ± 174	355 ± 169	362 ± 185	373 ± 216	348 ± 200	357 ± 209
**Creatinine (mg/dL)**	0·4 ± 0·2	0·4 ± 0·2	0·4 ± 0·2	0·5 ± 0·1	0·4 ± 0·2	0·5 ± 0·2	0·5 ± 0·2
**ALT (IU/L)**	23 ± 33	23 ± 14	23 ± 24	20 ± 16	20 ± 12	24 ± 19	22 ± 16

Laboratory trends in the REACH trial, with results shown as mean ± standard deviation. Hb = haemoglobin, MCV = mean corpuscular volume, HbF = fetal haemoglobin, ANC = absolute neutrophil count, ARC = absolute reticulocyte count, ALT = alanine transferase. Children received fixed-dose hydroxyurea through Month 6, followed by dose escalation to MTD by Year 2. The latest values refers to the last laboratory parameters for 522 current participants, with treatment duration ranging from 75 to 108 months.

Clinical events	$-\alpha^{3.7}/\alpha\alpha$ (1-gene deletion) IRR (CI), p value	$-\alpha^{3.7}/-\alpha^{3.7}$ (2-gene deletion) IRR (CI), p value	G6PD deficient male IRR (CI), p value
**Painful events**	1.08 (0.98-1.18) 0.13	1.01 (0.87-1.18) 0.88	1.06 (0.9-1.26) 0.48
**Acute chest syndrome**	1.08 (0.76-1.54) 0.68	0.69 (0.35-1.35) 0.28	1.90 (1.03-3.51) 0.04
**Splenic sequestration**	2.48 (1.16-5.3) 0.02	0.84 (0.18-3.9) 0.83	1.64 (0.41-6.57) 0.48
**Stroke**	0.38 (0.15-0.91) 0.04	0.00 0.99	0.41 (0.05-3.67) 0.42
**Malaria**	0.91 (0.79-1.04) 0.17	0.73 (0.57-0.93) 0.02	1.13 (0.86-1.48) 0.36
**Non-malarial infections**	1.11 (1.03-1.19) 0.01	1.07 (0.95-1.2) 0.29	1.10 (0.96-1.26) 0.19
**SAE**	0.99 (0.71-1.37) 0.94	0.68 (0.37-1.25) 0.21	1.16 (0.63-2.17) 0.63
**Death**	0.82 (0.44-1.51) 0.52	0.54 (0.16-1.81) 0.32	1.64 (0.53-5.09) 0.39

Effects of alpha thalassemia trait status and G6PD deficiency on clinical events. For alpha thalassemia, 1 and 2 gene deletion is compared to wild type status. For G6PD deficiency, deficient males are compared to carrier females.

	Screening				Fixed Dose (reference)		Dose Escalation				MTD			
Study Month	-2 to 0				0 to 6		7 – 24				>24			
Patient-Years	111				299		878				3163			
Dose, mg/kg/day	--				17·5 ± 2·3		21·9 ± 5·0				24·5 ± 5·5			
Clinical Events	Events	Rate	IRR (CI)	p-value	Events	Rate	Events	Rate	IRR (CI)	p-value	Events	Rate	IRR (CI)	p-value
Painful Events	113	101·9	1·39 (1·11-1·75)	0·0037	217	72·7	338	38·5	0·53 (0·45-0·63)	<0·001	1377	43·6	0·60 (0·52-0·70)	<0·001
Acute Chest	10	9·0	0·90 (0·45-1·80)	0·76	30	10·0	39	4·4	0·44 (0·27-0·72)	0·0009	65	2·1	0·21 (0·13-0·33)	<0·001
Primary Stroke	0	0·0	--	--	1	0·35	3	0·36	1.02 (0.11-9.70)	0.99	5	0·18	0.52 (0.06-4.43)	0.55
Secondary Stroke	2	33·8	1.81 (0.48-6.79)	0.38	3	18·6	3	6·7	0.38 (0.10-1.42)	0.15	6	4·5	0.27 (0.07-1.06)	0.061
Splenic Sequestration	2	1·8	1.75 (0·29-10·53)	0·54	3	1·0	12	1·4	1·36 (0·44-4·24)	0·59	22	0·7	0·70 (0·20-2·46)	0·57
Malaria	52	46·9	1.41 (1·01-1·97)	0·044	98	32·8	196	22·3	0·68 (0·54-0·86)	0·0015	595	18·8	0·58 (0·46-0·72)	<0·001
Non-malarial infections	167	150·5	1.23 (1.02-1.47)	0·029	373	124·9	774	88·2	0·71 (0·63-0·80)	<0·001	2048	64·8	0·52 (0·46-0·58)	<0·001
Serious Adverse Events	12	10·8	1.32 (0·64-2·73)	0·45	23	7·7	36	4·1	0·55 (0·32-0·93)	0·025	97	3·1	0·42 (0·27-0·67)	0·0003
Death	4	3·6	2.72 (0·69-10·69)	0·15	4	1·3	10	1·1	0·84 (0·27-2·68)	0·77	30	0·9	0·70 (0·25-1·97)	0·50

Trends in clinical events based on hydroxyurea treatment dosing phase, presented as rates per 100 patient-years. The incidence rate ratio (IRR) and 95% Confidence Intervals (CI) compare the clinical event rates at each dosing phase to the fixed-dose period as the reference. MTD = maximum tolerated dose.

	**Screening**				**Fixed Dose (reference)**		**Dose Escalation**				**MTD**			
**Study Month**	-2 to 0				0 to 6		7 – 24				>24			
**Total CBC performed**	2,013				3,758		7,600				15,744			
**CBC with DLT (n, %)**	21 (1.0%)				55 (1·5%)		167 (2·2%)				569 (3·6%)			
**DLT found at sick visit (n, % of all DLTs)**	8 (38·1%)				12 (21·8%)		41 (24·6%)				179 (31·5%)			
**DLT type**	Events	Rate	IRR (CI)	p-value	Events	Rate	Events	Rate	IRR (CI)	p-value	Events	Rate	IRR (CI)	p-value
**Any DLT**	24	21.6	0.89 (0.52-1.51)	0.66	72	24·1	193	22.0	0.92 (0.65-1.31)	0.64	733	23.2	0.97 (0.70,1.35)	0.86
**Hemoglobin**	9	8.1	0.92 (0.42,1.99)	0.86	26	8·7	51	5.8	0.68 (0.41, 1.14)	0.14	188	5.9	0.70 (0.44, 1.12)	0.14
**Neutrophils**	1	0.9	0.29 (0.03, 2.48)	0.26	9	3.0	38	4.3	1.44 (0.70, 2.98)	0.32	123	3.9	1.30 (0.65, 2.60)	0.46
**Reticulocytes**	3	2.7	0.47 (0.14, 1.62)	0.24	17	5·7	46	5.2	0.92 (0.51, 1.66)	0.79	208	6.6	1.15 (0.69, 1.94)	0.59
**Platelets**	11	9.9	1.47 (0.69, 3.11)	0.32	20	6·7	58	6.6	0.99 (0.59, 1.66)	0.97	214	6.8	1.02 (0.59, 1.76)	0.95

Dose-limiting toxicities in the REACH cohort, by dosing phase and type of laboratory toxicity. DLT were protocol-defined for each laboratory parameter as described in the Methods. DLT events are shown as total number (n) in the dosing period and the rate per 100 patient-years. The incidence rate ratios (IRR), 95% confidence Intervals (CI) and p-values are for comparisons of rates in each dosing phase to the fixed-dose period as the reference.
